# Slow and steady wins the race: Spatial and stochastic processes and the failure of suppression gene drives

**DOI:** 10.1111/mec.16598

**Published:** 2022-07-22

**Authors:** Jeff F. Paril, Ben L. Phillips

**Affiliations:** ^1^ School of BioSciences University of Melbourne Parkville Victoria Australia

**Keywords:** gene drive chasing, invasion waves, spatial simulations, suppression gene drive, W‐shredder, X‐shredder

## Abstract

Gene drives that skew sex ratios offer a new management tool to suppress or eradicate pest populations. Early models and empirical work suggest that these suppression drives can completely eradicate well‐mixed populations, but models that incorporate stochasticity and space (i.e. drift and recolonization events) often result in loss or failure of the drive. We developed a stochastic model to examine these processes in a simple one‐dimensional space. This simple space allows us to map the events and outcomes that emerged and examine how properties of the drive's wave of invasion affect outcomes. Our simulations, across a biologically realistic section of parameter space, suggest that drive failure might be a common outcome in spatially explicit, stochastic systems, and that properties of the drive wave appear to mediate outcomes. Surprisingly, the drives that would be considered fittest in an aspatial model were strongly associated with failure in the spatial setting. The fittest drives cause relatively fast moving, and narrow waves that have a high chance of being penetrated by wild‐types (WTs) leading to WT recolonization, leading to failure. Our results also show that high rates of dispersal reduce the chance of failure because drive waves get disproportionately wider than WT waves as dispersal rates increase. Overall, wide, slow‐moving drive waves were much less prone to failure. Our results point to the complexity inherent in using a genetic system to effect demographic outcomes and speak to a clear need for ecological and evolutionary modelling to inform the drive design process.

## 1 INTRODUCTION

The discovery of CRISPR‐Cas9 will soon revolutionize our capacity to manipulate populations. By enabling precise gene editing of individuals, CRISPR‐Cas9 allows us to introduce novel genes into populations (Chang et al., 2013; DiCarlo et al., 2013; Friedland et al., 2013; Horvath & Barrangou, 2013; Kyrou et al., 2018; Miao et al., 2013; Shen et al., 2013; Yu et al., 2013). Such novel genes are, like all functional genes, subject to natural selection. But by pairing the novel genes we wish to introduce with selfish genetic elements, we can introduce genetic constructs that will tend to increase in frequency despite fitness costs. Such ‘gene drives’ allow us to drive traits into populations, against the force of natural selection (Noble et al., 2017). In principle, we can use such constructs to change the very demographics of a population: to reduce population fitness, and even to drive populations extinct (Hammond et al., 2021; Kyrou et al., 2018).

The possibility that we might use gene drives for population suppression is of clear interest in the management of pests and diseases. Globally, we spend billions of dollars annually to the control of pests and diseases (e.g. annual ryegrass and rodents, Bradshaw et al., 2021), and many of the tools we use are blunt ones: they involve environmental impacts and raise ethical questions (Hough, 2021). Gene drives offer a new alternative. Various suppression gene drive systems have now been proposed, most of which make use of CRISPR‐Cas9 (Champer et al., 2020; Champer, Champer, et al., 2021; Macias et al., 2017; Sinkins & Gould, 2006). These gene drives aim to eradicate populations by forcing the inheritance of fitness‐reducing alleles leading to sex ratio distortion, sterility and/or lethality. Such systems, in principle, offer highly targeted control and completely avoid the environmental and welfare impacts of many existing pest control strategies.

There remains substantial work to be done to develop effective, safe, targeted and socially acceptable control options using gene drives. On the technical front, an effective gene drive needs to invade the target population and attain a frequency high enough to effect management aims. To do so, it will need to push hard against the force of natural selection, that is, it needs to reach target frequencies despite fitness costs and the emergence of resistance alleles. This is not a trivial challenge, but several constructs now exist that prevent drive resistant alleles emerging within short time scales under laboratory conditions, and which can be used to skew sex ratios until the population collapses. Such constructs work in theory and have also been shown to drive laboratory populations extinct. For example, CRISPR‐Cas9 gene drives have driven caged populations of *Anopheles gambiae* mosquitoes to extinction (Kyrou et al., 2018; Simoni et al., 2020) and have also been demonstrated to cause significant sex distortion in *Ceratis capitata* fruit flies under controlled laboratory conditions (Meccariello et al., 2021). These laboratory trials are encouraging proofs of concept, but whether such constructs will work in a real setting remains to be seen.

One of the major challenges to the effectiveness of drive constructs for population suppression arises when we move these systems from the laboratory and into more realistic spatially explicit settings. Here, the very success of a construct might work against it. In a spatially explicit setting, population suppression at one location immediately sets up a density gradient between suppressed and non‐suppressed areas. This gradient causes an asymmetry in gene flow, such that there is a net flow of genes from non‐suppressed areas into the suppressed areas. Thus, in space, a drive construct needs to push against both natural selection, and gene flow (Girardin et al., 2019).

To guide our understanding of how gene drives might behave in real settings, analytical and simulation models are valuable tools. The earliest gene drive models take the sensible simplifying step of ignoring space: they assume panmixia. These mathematical models and population‐based stochastic simulations routinely show that suppression gene drives will rapidly crash populations to extinction (Burt & Deredec, 2018; Deredec et al., 2008; Prowse et al., 2017). But when we introduce space, and stochastic processes, things become complicated. The clearest stochastic evolutionary process is drift, in which alleles can be lost from small populations despite deterministic expectations that they should increase in frequency. When space is included in models, we also have to contend with founder events, which are essentially a spatial manifestation of drift (Slatkin & Excoffier, 2012). These two processes can lead to surprising emergent dynamics that are absent from deterministic and aspatial models. A founder event in which drive alleles have been lost can cause a recolonization event in which previously cleared space is recolonized by wild‐type (WT) individuals. When such events are sufficiently common, the result can be a complex dynamic in which the gene drive endlessly chases the WT population and so fails to effect eradication (Birand et al., 2022; Champer, Kim, et al., 2021; Liu & Champer, 2022). A continuous space is not a requirement for this chasing to occur: the phenomenon has been observed in simulations of linked discrete populations and in other highly structured populations (Bull et al., 2019; North et al., 2019).

With spatial and stochastic processes in play, we take a drive system that is capable of crashing a large, panmictic population, and we observe it fail to crash a finite, spatially structured population. If we are ever to apply gene drive technology to the control of pest species, it is clear that we need to understand these stochastic and spatial effects. What are the conditions under which they manifest? Are some drive systems less susceptible to spatial effects than others? Answering these questions will bring us a step closer to the effective control of spatially structured populations such as cane toads in Australia (Urban et al., 2008), and lepidopteran pest populations in large‐acre farming (Anderson et al., 2016; Jones et al., 2019).

While analytical models often offer more general conclusions, they rapidly become intractable when bent to describe gene drives with demographic effects (Girardin & Débarre, 2021), and become more complex still with the inclusion of stochasticity. In this paper, we gain some insight from analytical models, but largely approach from the opposite direction, with an individual‐based simulation model in which complex dynamics can emerge. We sacrifice general conclusions in favour of phenomenological description of emergent patterns. In our model, we consider the waves of invasion of both WT and gene‐drive populations. We commence with the expectation that there are situations (i.e. parts of parameter and model space) in which stochastic effects are negligible, and situations in which they are not. Failure typically does not occur where stochastic effects are negligible. Importantly, the impact of stochastic effects is in large part controlled by two characteristics of the emergent invasion waves. First, the width of these waves matter: relatively narrow drive waves are more likely to drift to extinction or to be penetrated by WTs than are wide drive waves. Second, once a drive wave has been penetrated, the relative velocity of the drive wave matters: drive waves that move faster than WT waves are more prone to drive loss following recolonization events. Both wave shape and velocity are strongly affected by the choice of the suppression drive system. Thus, careful choice of drive systems can avoid the worst stochastic outcomes by generating a relatively wide invasion wave whose velocity is calibrated to be similar to that of the WT.

## 2 SIMULATION METHODS

We performed spatially explicit individual‐based stochastic simulations of suppression gene drive introduction to eradicate a WT population. We complement these simulations with the derivation of the recursive gene drive allele frequency equations for the most well‐studied suppression gene drive systems, the sex distorter drives: X‐shredder and W‐shredder (Burt & Deredec, 2018; Galizi et al., 2016; Holman, 2019; Prowse et al., 2017; Simoni et al., 2020). From these equations, we built naive partial differential equations (PDEs; which ignore advection, i.e. the bulk movement of alleles in a particular direction, caused by asymmetric gene flow) describing gene drive and WT dynamics across one‐dimensional (1D) space, and derived Fisher's asymptotic spreading velocities for the two suppression gene drive systems. Because advection is ignored, these Fisher velocities can be thought of as the maximum velocity that a drive wave might achieve.

### 2.1 Local dynamics

We simulated a population with discrete non‐overlapping generations, inhabiting a continuous 1D space with a total length of 2500 on a unitless scale. This bounded 1D space has absorbing boundaries simulating the phenomenon where individuals dispersing beyond their range fail to survive. Bounded space is a pragmatic choice in that it reflects the reality of species ranges. It is important to note that some of the pathways to drive success depend upon this feature of space (see ‘Emergent dynamics’ below).

Individuals are assumed to spread their density across space according to a univariate normal distribution with standard deviation of one. This distribution of density can be interpreted as area of influence of each individual in the continuous space. Local density experienced by an individual, $N_{i}$ is calculated as the sum of densities across all individuals at the reference individual's location:

(1) $$N_{i}=\sum_{j=1}^{n}\frac{1}{\sqrt{2\pi }}e^{-d_{i,j}^{2}/2},$$

where $d_{\mathrm{ij}}$ is the Euclidean distance between individuals i and j, and n is the total number of individuals in the population at any given time.

Females choose a mate from their local neighbourhood, within three spatial units. This constraint provides a spatial Allee effect: individuals that cannot find a mate die without reproducing. A male within this range is chosen at random, but the probability of choosing a particular male scales with the density that male provides to the female's location (given by the individual density described in Equation (1)). Mated females produce offspring with an expected reproductive output defined by the Beverton–Holt growth function:

(2) $$E\left(W_{i}\right)=\frac{R}{1+{\text{aN}}_{i}},$$

where R is the maximum expected number of offspring a female can produce which ranges from 2 to 10, and a determines the response to density. In our case, we are modelling discrete sexes with an expected equal sex ratio, so we set $a=\frac{R-2}{2N^{*}}$, where $N^{*}$ denotes the equilibrium population density per spatial unit and was set to 5 in all simulations. A female's realized number of offspring is given by a draw from a Poisson distribution, $W_{i}∼\text{Poiss}\left(E\left(W_{i}\right)\right)$.

### 2.2 Dispersal dynamics

Offspring are born at the mother's location and immediately disperse. Dispersal is described by a univariate normal distribution with standard deviation, $\sigma \in \left[2,20\right]$. This dispersion variable defines the root mean squared dispersal distance as σ spatial units in any given simulation. Thus, σ sets the scale of dispersal (relative to the scale of local dynamics) in each simulation. This range translates to an average movement of between 0.1% and 1% of the landscape's width per individual per generation, which is within range of empirically measured dispersal rates of many established invasive species, for example, cane toads (Kearney et al., 2008; Phillips et al., 2006; Urban et al., 2008).

### 2.3 Gene drives and population suppression

We studied two sex‐distorter suppression gene drives, X‐shredder and W‐shredder. X‐shredder works on the XY sex determination system, where individuals with XX chromosomes are females while those with XY are males. On the other hand, W‐shredder works on the ZW sex determination system, where ZW individuals are females and ZZ individuals are males. Both these systems have the ability to eradicate populations by skewing the sex ratio in favour of males, hence reducing the overall fecundity of the population until the population becomes 100% male and becomes extinct.

In the X‐shredder system, the Y chromosome carries the homing endonuclease gene drive (Y^d^) which creates breaks along the X chromosome rendering it dysfunctional. As a consequence, XY^d^ males will only produce Y^d^ gametes. We assume the fecundity or fertility of male drive‐carriers are unaffected, that is, a female mating with males with shredded or unshredded X chromosomes are equally likely to bear offspring. This X‐shredder system is also referred to as ‘Driving Y X‐shredder’ since the homing endonuclease gene drive is located on the Y chromosome and not on autosomes. Similarly in W‐shredder, the Z chromosome carries the homing endonuclease gene drive (Z^d^) which likewise ‘shreds’ the W chromosome. As a consequence, Z^d^W females will only produce Z^d^ gametes. We also assume that the Z^d^W females have unaltered fecundity. This W‐shredder system is also referred to as the ‘Z‐linked W‐shredder’ to stress the location of the homing endonuclease gene drive on the Z chromosome.

For both W‐ and X‐shredders, we assume 100% shredding efficiency. We also assume that the gene drive alleles do not reduce the fecundity or the total gametic output of the individuals carrying them. Additionally, we ignore the possibility of evolution of resistance to shredding through mutated shredding target sites. Thus, we modelled an ideal gene drive system (which is yet to be achieved in the laboratory or in the field).

### 2.4 Simulated scenarios

For each of our drive types, we simulated a total of 100 different scenarios, each replicated 100 times. Each scenario is a combination of the two variables: maximum female fecundity, $R_{i}\in \left[2,10\right]$ and dispersion parameter, $\sigma_{i}\in \left[2,20\right]$ for i∈1,10 (10 equidistant points for each variable). In all scenarios, we set $N^{*}=5$. This sets our equilibrium population size across the entire spatial domain (2500 units) to 12,500 individuals. Within the more limited area defining local population dynamics (within three units of a focal individual), this carrying capacity implies a maximum local population size of approximately 30 individuals.

We initialized the WT population to uniformly occupy space between positions x=250 to x=1250 (10%–50% of the spatial domain). The suppression gene drive‐carriers (i.e. heterozygote for the drive allele) were introduced at 1% of the WT population size. These drive‐carriers were all placed at x=250, that is, the left‐most border of the initialized population. This initialization established two invasion waves: one for the WT population invading x>1250, and the other for the drive invading x>250. This enabled the reliable measurement of the properties of these waves, as defined below. Population dynamics proceeded for at most 1000 generations, or until the population was eradicated, or after an additional 10 generations following the loss of the drive allele, whichever occurred first. At each time step, we recorded the following:

- population size,
- proportion of females,
- population density (mean of the local densities of each individual),
- standard deviation of the population density (over space),
- drive allele frequency and
- mean number of offspring (total, and separately for both WT and drive‐carrier parents).

Additionally, the following wave properties were measured for both WT and drive waves before the waves reached the edge of the landscape and before drive wave penetration occurred:

- wave velocities: defined as the difference in location between the furthest leading individuals between t and t−1 (averaged across generations),
- wave heights: defined as the maximum density of the respective wave, that is, the wave peak (measured after one generation of mating and dispersion following gene drive introduction) and
- wave widths (measured after one generation of mating and dispersion following gene drive introduction): *WT wave width* defined as twice the distance between the farthest individuals and the peak of the wave at the leading edge; *drive wave leading half width*: distance between the leading individual and the peak of the wave; and *drive wave trailing half width*: distance between the trailing individual and the peak of the wave; *drive wave width*: *drive wave leading half width* plus *drive wave trailing half width*.

### 2.5 Emergent dynamics and outcomes

**FIGURE 1:**
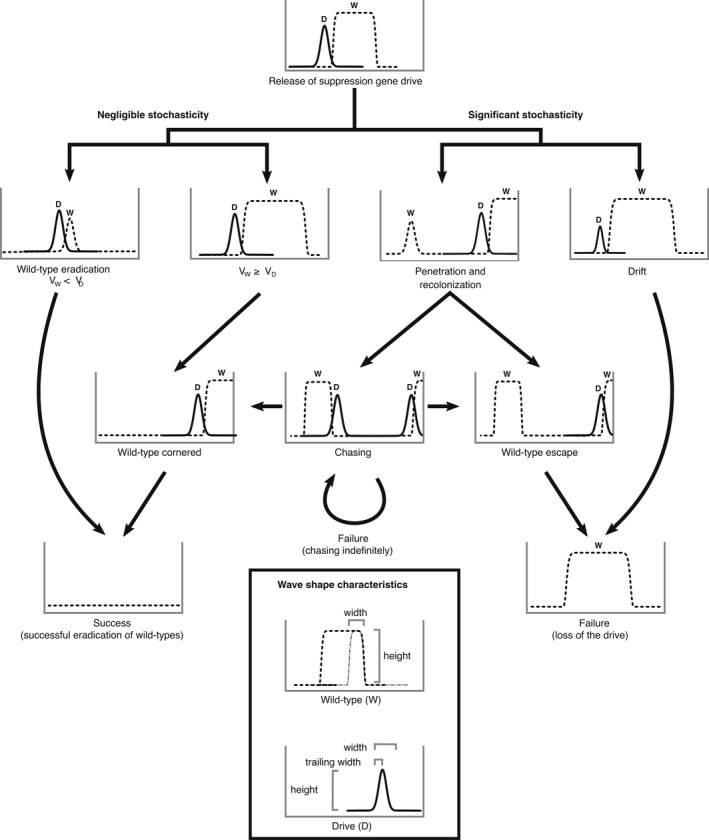
Schematic illustration of the events and outcomes of a spatially explicit individual‐based stochastic simulation of suppression gene drives. Figure panels show population density through space for both wild‐type (W) and gene drive (D) alleles at a snapshot in time. In a bounded one‐dimensional space, the simulation events which occur prior to the success or failure of the drive can be classified by characteristics of relative wave velocity, and the stochastic events of drift and recolonization. Our simulations involve an absorbing boundary such that the wild‐type population can be cornered by the drive wave. The box at the bottom illustrates how the wave shape characteristics, that is, wave heights and widths, were measured for the wild‐type and drive waves. The dashed‐line in the wild‐type curve represent the full width of the wild‐type population, while the dash‐dotted line represents the travelling wave measured as twice the distance between the farthest individuals and the peak of the wave at the leading edge.

We define suppression gene drive ‘success’ as the complete eradication of the WT population on or before t=1000 generations. ‘Failure’ is defined as the population being extant at t=1000, which may be due to the loss of the drive or to the coexistence of both WT and gene drive alleles. With regard to loss of the drive, there are two pathways by which this might happen (Figure 1). First, in an event we call ‘drift loss’, the drive might be lost very early in the simulation through drift. This is a global loss of the drive and leads to immediate failure. The other process through which we might see loss of the drive is a recolonization event, in which a drive‐free population recolonizes space recently cleared by the suppression drive. This initially only causes local loss of the drive but has several possible downstream outcomes. Local loss might lead to global loss in an event we call ‘WT escape’. Here, the recolonization event leaves a WT population isolated in space from the drive population. The drive then goes on to eradicate the remainder of the WT population and then goes extinct itself, leaving the isolated population to recolonize empty space. This outcome should be particularly likely if the drive wave is faster than the WT wave. Where the drive wave is slower than the WT wave, we often see ‘drive chasing’ in which a recolonizing WT population is reinvaded by the drive, causing the drive wave to spread in both directions. In some parts of parameter space, this occurred only occasionally and so would lead to eradication as the drive corners the WT against the spatial boundaries of the population. In many parts of parameter space, however, founder and recolonization events were frequent enough to allow the population to persist to t=1000. For purposes of scoring our various simulations, we define chasing as the reinvasion of the drive following drive‐wave penetration by WTs. We scored drive wave penetration by WTs as occurring if at least one WT individual moved at least σ units beyond the trailing edge of the gene drive wave (defined as the drive‐carrying individual with the lowest value of x). The ability of this procedure to detect penetration hinges upon the presence of a single WT wave and will fail if multiple WT waves have emerged; thus, it only reliably measures the first penetration event. Additionally, it is important to point out that scenarios which resemble WT cornering or escape can transition towards indefinite chasing, drive loss and population eradication, since these are all subject to the stochasticity of the simulations. These events and outcomes are depicted in Figure 1.

## 3 HOW OFTEN DO WE EXPECT THESE SUPPRESSION GENE DRIVES TO FAIL? AND WHY DO THEY FAIL?

Across the parameter space we explored, we found that the suppression gene drives, X‐shredder and W‐shredder, failed to eradicate the WT population in more than half (50.7%; 10,132/20,000) of our simulations. The X‐shredder drive was notably more prone to failure (58%; 5750/10,000) than W‐shredder (44%; 4382/10,000). This is a striking failure rate: under an aspatial context (or well‐mixed populations) with the same 100% conversion efficiency and the impossibility of resistance evolution, both drives will successfully eradicate the WT population 100% of the time (excluding drive loss due to drift).

**FIGURE 2:**
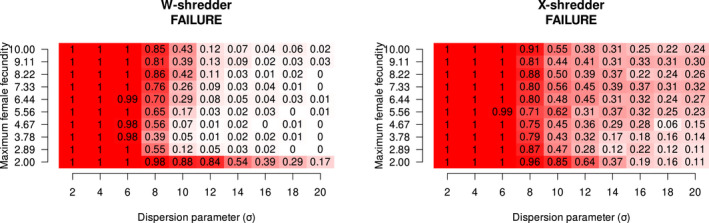
Estimated probability of failure of W‐shredder and X‐shredder suppression gene drives to eradicate the target population. Failure probability is shown as a function of the maximum female fecundity and dispersion parameters.

In Figure 2, we present the estimated probabilities of success and failure of the gene drive across our $\left\{R_{\max},\sigma \right\}$ parameter space, examining the effect of maximum female fecundity and dispersion parameter on failure probability. Failure becomes less likely as dispersion increases. This is expected because populations approach panmixia as dispersion increases (we are moving towards an aspatial system), but failures appear to become rare well before panmixis is achieved. The range of maximum female fecundity we tested appeared to have a weaker effect on the outcomes; failure tended to be more likely at the lowest extreme of maximum growth rate, that is, $R_{\max}=2$. This agrees with the previous results of Champer, Kim, et al. (2021). At no growth ($R_{\max}=2$), failure is markedly more likely even at the upper range of the dispersion parameter, because drive‐carriers cannot produce enough offspring to prevent loss due to drift in sparsely populated areas. Otherwise, there is a slight tendency towards increased failure as growth rate increases. Drive failure is notably more common in X‐shredder than W‐shredder. These relationships are expressed concisely in Figure S1 as violin plots, where we assumed completely additive effects of the drive type, maximum female fecundity and dispersion parameter.

These broad results raise a number of questions. These are drives that, in an aspatial simulation, will send the population extinct. Why are they failing at such a high rate in this spatial setting? Why did growth rate or female fecundity only weakly affect the success of the drive? What properties differentiate W‐shredder from X‐shredder so much so that success rates between them are significantly different? In the following, we attempt to answer these questions and identify the characteristics of an ideal suppression gene drive. Finally, we put forth recommendations on key areas of study required before we can successfully use suppression gene drives to control invasive species, pests and diseases.

**FIGURE 3:**
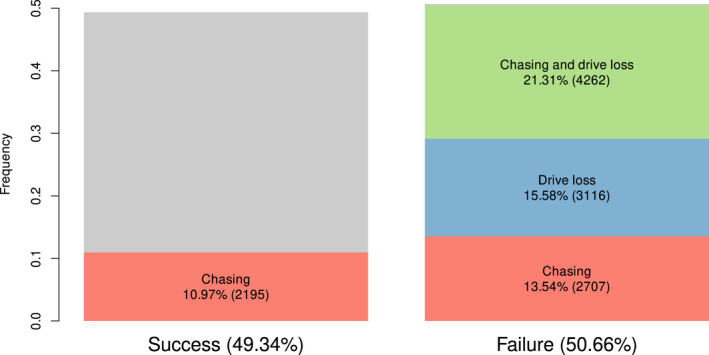
Frequencies of the simulation outcomes (suppression gene drive success and failure), and their intersections with the events that can cause failure: Loss of suppression gene drive allele and chasing.

The fundamental reasons for the failure of suppression gene drives to eradicate the population can be broadly divided into two: indefinite chasing and drive loss. Drive loss (i.e. loss of drive alleles despite the presence of WTs) can occur through either drift early in the simulation or through WT escape following a later recolonization event (Figure 3). Drive loss must always result in the failure of the drive, and it occurred in 36.89% (7378/20,000) of our simulations. The remaining failures we observed were from populations reaching 1000 generations with both WT and drive alleles still in coexistence. These failures are caused by the complex chasing dynamic in which recolonization events are sufficiently common that cornering never happens; chasing continues indefinitely. The necessary event for nearly all these failure types is chasing; an outcome that occurred in 68.79% of our simulations which resulted in failure. We also showed that the higher failure rate in X‐shredder compared with W‐shredder is attributable to the higher frequency of drive loss with and without prior chasing (Figure S2).

Under our definition, chasing occurs if the drive reinvades a recolonizing WT population. Where founder and recolonization events are common, the process of extinction is continuously undermined by local WT escapes. This can lead to a dynamic in which global coexistence occurs despite complete local turnover of allele frequencies; a situation that occurred in 13.54% (2754/20,000) of our simulations. Deterministic models of suppression gene drives do not deliver this dynamic (Beaghton et al., 2016; Tanaka et al., 2017). Champer, Kim, et al. (2021) reported this phenomenon in both 2D and 1D space, but the characteristics of the travelling population waves were not studied in detail. It is clear that this global coexistence is a fundamentally stochastic phenomenon rooted in founder events occurring beyond the retreating population margin.

## 4 RELATIVE WAVE VELOCITY AND CHASING

Although we imagine a bounded system, allowing WTs to be cornered by the drive, it is clear that the relative speeds of the WT and drive waves may be important. In particular, they are likely to be important following a founder‐induced local loss of the drive. Under some assumptions, it is possible to derive the expected speeds from a deterministic model, and we examine this now to see what insight can be drawn. The mathematical models used in these derivations are deterministic. Drive loss due to drift and recolonization cannot occur. Furthermore, the drive wave also assumes homogeneous population density (i.e. it does not account for advection—the asymmetric gene flow caused by suppression—and so will be an upper bound on the drive speed). We derive these analytic speeds and compare our results to stochastic simulations in which we measured the wave velocities of the WTs and drive‐carriers.

### 4.1 Velocities of WT and drive waves

We described population density and allele frequency dynamics across time and 1D space. We derived the velocities of the WTs invading empty space, and the suppression gene drive alleles invading the WT population using a system of PDEs. To define these PDEs, we derived the recursive allele frequency functions, $q_{t+1}=f\left(q_{t}\right)$ for W‐shredder and X‐shredder, as well as the recursive population density function, $n_{t+1}=g\left(n_{t}\right)$. These functions were used to define the PDEs assuming overlapping generations (continuous time), that is, $\frac{dq}{dt}=\Delta q=q_{t+1}-q_{t}$ and $\frac{dN}{dt}=\Delta N=N_{t+1}-N_{t}$. The advection term (Girardin et al., 2019; Girardin & Débarre, 2021) in the PDE for drive allele frequency was ignored because it massively complicates the calculation of velocity. Ignoring this term is effectively ignoring asymmetrical gene flow caused by the density gradient arising from suppression and so in this case yields a velocity that is higher than the velocity that would be realized if we accounted for asymmetrical gene flow. Without the advection term, we can calculate velocities using Fisher's definition of asymptotic spreading speed (Fisher, 1937; Lewis et al., 2016).

We derived the recursive suppression gene drive allele frequencies, $q_{t+1}$ for W‐shredder to be:

(3) $$q_{t+1}=\frac{8q_{t}-3{\mathrm{cq}}_{t}-{\mathrm{cq}}_{t}^{2}}{8-4c},$$

and for X‐shredder it is:

(4) $$q_{t+1}=\frac{2q_{t}}{2-c+{\mathrm{cq}}_{t}},$$

where c is the chromosomal shredding efficiency of the gene drive (refer to Supporting Information for details of the derivations, i.e., subsections ‘Derivation of W‐shredder equations’ and ‘Derivation of X‐shredder equations’).

**FIGURE 4:**
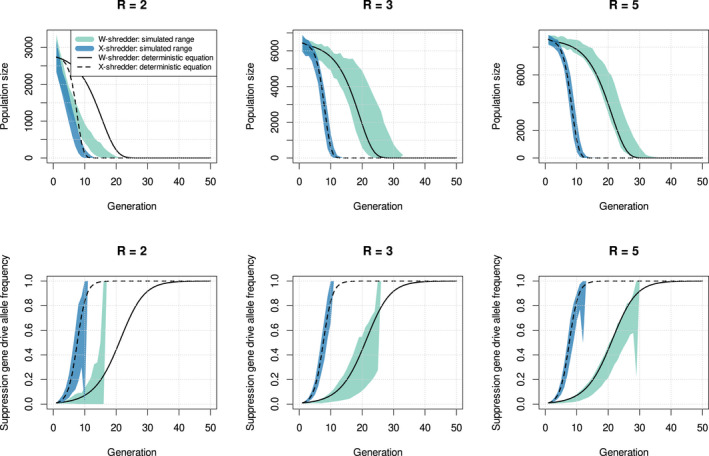
Time‐series plots of total population size and suppression gene drive allele frequency across generations using the stochastic simulation data and the deterministic recursive equations for W‐shredder and X‐shredder suppression gene drives. R is the maximum female fecundity, and the dispersion parameter was set to σ=10. The bands of green (W‐shredder) and blue (X‐shredder) represent the simulated ranges of population sizes and suppression gene drive allele frequencies. The solid (W‐shredder) and dashed (X‐shredder) lines represent the deterministic recursive equations for population size and drive allele frequency.

We derived the recursive equation for population density, $N_{t+1}$ to be:

(5) $$N_{t+1}=N_{t}\phi_{t}b_{t},$$

where the frequency of females at generation t is $\phi_{t}=\frac{2-c-{\mathrm{cq}}_{t-1}}{4-2c}$, and the female fecundity, $b_{t}$ is the Beverton–Holt growth function (Equation (2)). These deterministic recursive equations approximate the output of the stochastic simulations under approximate panmixia (Figure 4). However, the deterministic equations have a tendency to overestimate the rates of decrease in population size and the rates of increase in drive allele frequencies. This is largely what we would expect given the lack of accounting for advection, but Figure 4 gives us a sense that the bias is mild relative to the variance introduced by stochasticity.

To derive the asymptotic gene drive and WT velocities, we assumed overlapping generations (continuous time) yielding the gene drive allele frequency differential for W‐shredder as:

(6) $$\frac{dq}{dt}=\frac{\mathrm{cq}\left(1-q\right)}{8-4c},$$

and for X‐shredder as:

(7) $$\frac{dq}{dt}=\frac{\mathrm{cq}\left(1-q\right)}{2-c+\mathrm{cq}}.$$

These results show that X‐shredder has a higher rate of increase than W‐shredder; in this sense, it is the ‘intrinsically fitter’ of the two drives.

When we assume overlapping generations or continuous time, the Beverton–Holt function is analogous to the logistic growth function (Bohner & Warth, 2007):

(8) $$\frac{dN}{dt}=\mathrm{rN}\left(1-\frac{N}{K}\right),$$

where r is the intrinsic growth rate (also called the low‐density growth rate) and K is the carrying capacity (the equilibrium density of individuals along the spatial *x*‐axis, in the absence of suppression and migration).

So far, these equations (Equations ((3), (4), (5), (6), (7), (8))) are not spatially explicit. To account for the spatial dynamics, we formulated the PDE for population density $\frac{\partial N}{\partial t}$ in 1D space to be:

(9) $$\frac{\partial N}{\partial t}=D\frac{\partial^{2}N}{\partial x^{2}}+\text{growth rate}+\text{suppression rate},$$

where

(10) $$\text{growth rate}=\frac{dN}{dt},$$

D is the diffusion coefficient. We can calculate the velocity by examining parts of space a long way from the invasion front, where $\frac{N}{K}\approx 0$ (low density) and suppressionrate≈0, then the PDE simplifies to:

(11) $$\frac{\partial N}{\partial t}=D\frac{\partial^{2}N}{\partial x^{2}}+\mathrm{rN}.$$

The asymptotic velocity (as t→∞) of the WT individuals in occupying the open space is therefore:

(12) $$v_{\text{wild type}}=2\sqrt{\mathrm{Dr}}.$$

Similar logic can be used to derive the asymptotic velocity of the invading suppression gene drive using Fisher's reaction–diffusion equation (Fisher, 1937). We need to make an additional assumption that the density gradient resulting from the suppression drive is effectively zero in the vicinity of the leading edge of the gene drive invasion. Ignoring the advection term effectively gives us an upper bound on the gene drive velocity of:

(13) $${\hat{v}}_{\text{gene drive allele}}=2\sqrt{\mathrm{Dm}},$$

where m is the selective advantage of the drive, but since fitness cost was not simulated, we interpret this as the intrinsic fitness of the drive. This intrinsic fitness represents the overall ability of drive to increase in frequency, which is different from the canonical definition as the reproductive potential of the drive‐carrier. In W‐shredder this is:
(14)

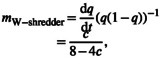

and in X‐shredder this is:
(15)

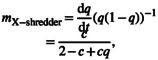

since $\frac{dq}{dt}=\mathrm{mq}\left(1-q\right)$. The intrinsic fitness of W‐shredder does not depend on q while X‐shredder does, because the drive spreads with both males and females in ZW mating systems and only in males in XY mating systems. This yields the upper bound velocity of the W‐shredder drive as:

(16) $${\hat{v}}_{W‐\text{shredder}}=2\sqrt{\frac{D}{4}},$$

and the upper bound velocity of the X‐shredder assuming q≈0 (i.e. very low frequency at the wave front) as:

(17) $${\hat{v}}_{X‐\text{shredder}}=2\sqrt{D}.$$

Thus, we find that the WTs are faster than the suppression gene drives at $r>\frac{1}{4}$ for W‐shredder, and at r>1 for X‐shredder. Mapping the low‐density growth rate, r into the non‐overlapping case, assuming non‐skewed sex ratio, ϕ=1/2 and low‐density such that $b=R_{\max}$, then:
(18)

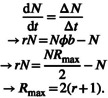




Hence, if the maximum female fecundity, $R_{\max}$ is greater than 2.5 for W‐shredder and greater than 4 for X‐shredder, then the WT wave is expected to be faster than gene drive. We note that these critical values of $R_{\max}$ do not appear to have a major impact on the probability of failure when viewed across all simulations (Figure 2).

In addition to the intrinsic drive fitness, the growth rate and dispersion rate also affect drive wave properties, but these are constant across WTs and drive‐carriers within simulations. The dispersion parameter (σ) is positively correlated with the width of waves, hence in comparing this wave characteristic across simulations, we used wave width relative to the dispersion parameter, that is, $\frac{\text{wave width}}{\sigma }$. The dispersion parameter also affects wave velocity but since we are comparing WT against drive wave and both are affected equally by the same σ, we instead use the velocity of the drive wave relative to the WT wave, that is, $v_{\text{drive}}-v_{\mathrm{WT}}$.

### 4.2 Penetrating the wave of invading drives

**FIGURE 5:**
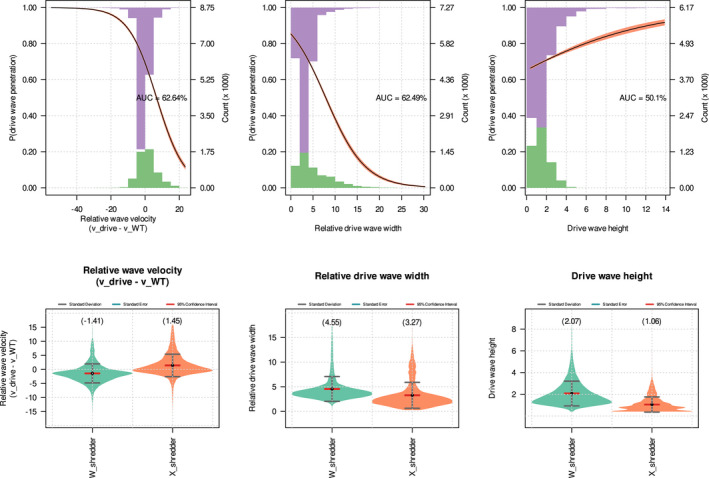
Suppression gene drive wave characteristics. Top panels: Logistic regression plots showing the relationship between the probability of wild‐types (WTs) penetrating the wave of suppression gene drive‐carriers and the three wave characteristics, that is, relative wave velocity (drive velocity − WT velocity), width of the drive wave relative to the dispersion parameter, σ (i.e. width/σ) and height of the drive wave. The purple and green histograms represent the distribution of presence and absence of drive wave penetration, respectively, across the range of wave characteristics. AUC refers to the area under the receiver operating characteristic curve, that is, true‐positive rate plotted against false‐positive rate, hence higher AUC means better logistic model fit. Bottom panels: Violin plots of the wave characteristics of W‐shredder and X‐shredder suppression gene drives. Grey bars indicate 1 standard deviation from the mean, while the red bars show to the 95% confidence interval.

While the relative velocity of the drive wave is reasonably easy to identify, the conditions promoting penetration and recolonization by WTs are less clear. The likelihood of a founder event causing local loss of drive alleles might depend upon both the velocity and shape of the gene drive wave front. All else being equal, a drive front that is slower than the WT, and very narrow should be more easily penetrated than one that is fast and wide. Indeed, we observed this in our simulations (Figure 5 top‐left and top‐centre panels): the probability of WTs penetrating the drive wave (simply referred to as ‘penetration’) was negatively correlated with the relative wave velocity ($v_{\text{drive}}-v_{\mathrm{WT}}$) and the width of the drive wave relative to the dispersion parameter (width/σ). By contrast, the wave height is not as important. We used relative wave velocities and relative wave widths to remove bias between the WT and drive waves and across varying dispersion parameters, respectively.

The violin plots in Figure 5 bottom panels show that the X‐shredder wave is faster than the W‐shredder wave (as expected from their respective intrinsic drive fitnesses: Equations 14 and 15). The higher intrinsic fitness of X‐shredder also results in thinner and slower waves than W‐shredder. Despite being slower, W‐shredder is less likely to result in drive failure after penetration (Figure S3 top left violin plot). This suggests that in bounded space, the ability of the drive wave to resist penetration is a more significant factor to drive success than the velocity of the drive wave. The thicker wave of W‐shredder compared with that of X‐shredder is because $m_{X‐\text{shredder}}>m_{W‐\text{shredder}}$ (Equations 14 and 15), and assuming a standard Fisher wave, the wave width is predicted to be $\sqrt{D/m}$ (Fisher, 1937). Counter‐intuitively, then, the higher intrinsic fitness of the X‐shredder drive explains its overall higher failure rate, through higher rates of penetration.

**FIGURE 6:**
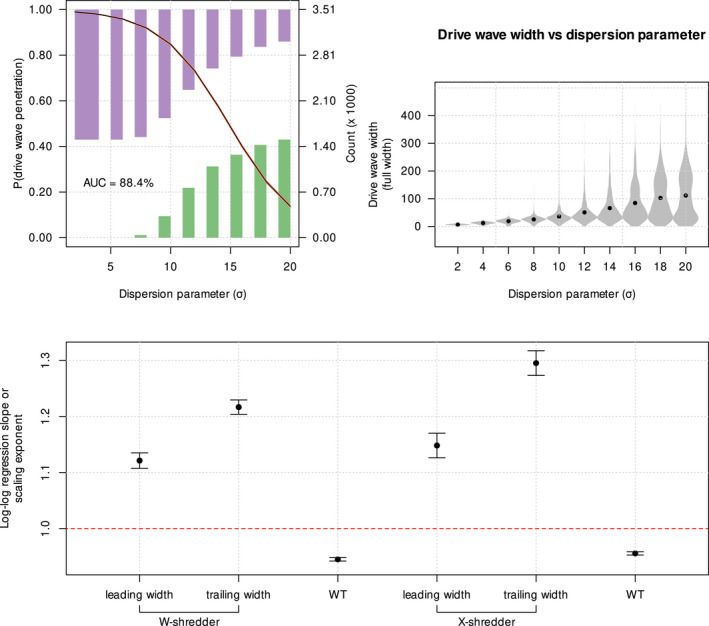
Relationships between the probability of suppression gene drive wave penetration by wild‐types, dispersion parameter and drive wave widths. Top‐left panel: Logistic regression plot relating the penetration probability with the dispersion parameter. The purple and green histograms represent the distribution of presence and absence of drive wave penetration, respectively, across the range of dispersion parameters. AUC refers to the area under the receiver operating characteristic curve, that is, the true‐positive rate plotted against false‐positive rate; a higher AUC means a better model fit. Top‐right panel: Violin plot of the full width of the drive wave across the dispersion parameter space. Log–log regression: $\ln\left(\text{width}\right)=a+s\ln\left(\sigma \right)$ was performed for the full width, leading half width and trailing half width of the gene drive wave. Bottom panel: Scaling exponents, s or the log–log regression slopes for the width of the wild‐type wave, as well as the trailing and leading widths of the suppression gene drive waves. Mean scaling exponents are shown as points, and error bars refer to ±1 standard error.

Naively, we might expect penetration to be unaffected by higher dispersion: an increase in dispersion might be expected to affect the WT and drive wave widths equally. But this is not what we observed. Indeed, the probability of penetration drops rapidly as dispersion increases, and this occurs at values of σ well below those approaching panmixia (Figure 6, top‐left panel). Assuming a Fisher wave, the WT wave should have a width equal to $\sqrt{D/r}$, where $D\propto \sigma^{2}$. Ignoring advection, the drive wave should have a width equal to $\sqrt{D/m}$. That is, we would naively expect both waves to scale linearly with σ. To examine this, we used a log–log regression to estimate the scaling exponent for each wave width against σ, where a scaling exponent of 1 means a linear relationship, while a scaling exponent >1 means an exponential relationship. We estimated the scaling exponent for the WT wave width to be 0.95 (±0.0021); sub‐linear, but approximately linear, as expected. By contrast, our simulations yielded scaling exponents of 1.22 (±0.0128) and 1.30 (±0.0221) for the widths of the trailing wave of W‐shredder and X‐shredder, respectively, additionally we estimated scaling exponents of 1.12 (±0.0138) and 1.15 (±0.0220) for the widths of the leading half of the W‐shredder and X‐shredder waves, respectively (Figure 6, bottom panel). This means that, relative to the WT, the drive waves get disproportionately wider as dispersion increases (Figure 6, top‐right panel). This result is almost certainly related to advection, that is, the asymmetric gene flow caused by the density gradient on the trailing edge of the population, and likely explains the rapid drop in penetration probability with increasing dispersion. In our model, σ sets the scale of dispersal relative to local dynamics; our results suggest that when the scale of dispersal is more than an order of magnitude greater than the scale of local dynamics, penetration rapidly becomes an unlikely event. The likely explanation for this transition is the super‐linear scaling of the drive width with σ. Whether our observation here is general, or a manifestation of the particular parameter space we examined, remains to be seen, but our results point to a fruitful avenue for further analytical exploration.

## 5 DRIVE LOSS

Loss of the suppression gene drive allele is a major reason why suppression gene drives fail to eradicate the target population, occurring in 37% of our simulations. Failure resulting from drive loss was most common in W‐shredder than X‐shredder (Figure S4). Drive loss results from drift and recolonization events, both of which are stochastic processes whose strength scales with population size. The probability of drive allele extinction through drift, P(drive loss | no penetration) is likely a function of the population size and the introduction frequency. In our simulations, we largely avoided this outcome using a relatively large number of introduced drive‐carriers (1% of the WT population size). As a consequence, early loss from drift occurred in only 16 simulations. Instead, most of the drive loss events we recorded occurred after penetration events. We would expect the probability of successful WT escape following recolonization events, P(drive loss | penetration) to be a function of population size as well as the velocity and width of the drive wave.

### 5.1 Drift: P(drive loss | no penetration)

Drift is the loss of an allele purely by chance. Its probability scales negatively with the increase in population size. Small populations are more likely to lose an allele than large ones. We confirmed this using our simulations by varying the equilibrium population density, $N^{*}$ from 2 to 5 (Figure S5). Furthermore, the frequency of the drive allele will be proportional to the likelihood of its fixation or loss. Low introduction frequency is more likely to result in drive loss than high initial frequency. Thus, drift loss can be controlled simply by introducing a sufficient number of drive‐carriers; it was a rare outcome in our simulations. Drift loss might also occur as the population is driven towards extinction by the drive, but we consider this less likely purely because the drive allele will be at very high frequency in this situation and so have a low likelihood of loss.

The effect of drift suggests that species or populations with high inbreeding to outbreeding ratio (e.g. self‐pollinating plants) are less likely to incorporate a newly introduced suppression gene drive allele unless the introduction frequency is a significant fraction of target population. Regardless of the inbreeding ratio, however, the density of a population plummets along the edge of the wave. Hence, populations are subject to stronger drift along their borders; deterministic equations will be poor guides to outcomes here.

### 5.2 WT escape: P(drive loss | penetration)

**FIGURE 7:**
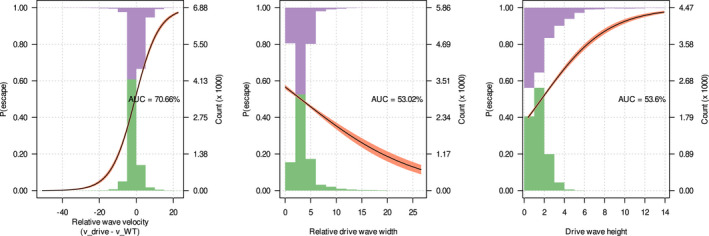
Logistic regression plot of the relationship between the probability of wild‐type escape (i.e. the probability of drive loss after drive wave penetration by wild‐types [WTs]) and the three wave characteristics, that is, relative wave velocity (drive velocity − WT velocity), width of the drive wave relative to the dispersion parameter, σ (i.e. width/σ) and height of the drive wave.

Founder events are the spatial equivalent of drift. Founder events can cause a drive wave to be penetrated by a founder population composed entirely of WTs. The likelihood that these founders will persist or crash is stochastic and a function of the founder size, hence the spatial analogue of drift. In many cases, penetration is followed by WT escape in which local loss of the drive through penetration becomes global loss following the recolonization of empty space by WTs and the extinction of the drive‐carrying population in other parts of space. Similar to its precursor event (penetration), escape likely depends on the velocity and the shape of the drive wave. In contrast to penetration, however, escape is more likely if the drive wave is faster than the WT wave (Figure 7 left plot). This stands to reason: if the drive wave is faster than the WT, then a newly formed WT population is unlikely to overtake the drive wave and so be reinvaded by it. Since faster drives generate thinner waves which are easier to penetrate than slower thicker ones, and given that 99.8% of the simulations which concluded with the loss of the gene drive allele were due to WT escape; it follows that slower drives are generally more effective; they decrease both the probability of penetration and the probability of subsequent WT escape.

Of course, after a penetration event, a complex chasing dynamic might ensue, which over multiple generations generates a complex landscape with multiple WT and drive waves growing, merging, dying and interacting with each other. During these scenarios, measuring wave characteristics became intractable. Exploring and discovering avenues to accurately measure these wave characteristics and interactions is an area we believe also worthy of future work.

## 6 CONCLUSION AND RECOMMENDATION

Our simulations are in agreement with those of others (Champer, Kim, et al., 2021): there is significant risk that suppression gene drives will fail in real‐world settings through spatial and stochastic processes. Previous stochastic models have revealed these processes in 2D spaces; we show that they are also present in the simplest 1D space, which was only previously hinted upon by Girardin and Débarre (2021). It is important to note that our results in 1D space may not directly extend to higher dimensional space; hence, an important question for future study is how does the probability of chasing scale with landscape dimensionality. Our simpler 1D space allows increased clarity as to the events and possible outcomes that emerge. It also allows us to focus on the fundamental aspects of the invasion waves – their width, height and speed – that are more difficult to measure in higher dimensional spaces. We show that these aspects of the drive and WT waves have a strong bearing on outcomes, but in a complex way. Overall, and somewhat counter‐intuitively, it is clear that slower and wider drive waves are substantially more effective than fast, steep waves. This contrasts very clearly with an aspatial view, in which faster (i.e. intrinsically fitter) drives will effect eradication more rapidly, and with lower chance of resistance evolving. However, we recognize that slower drives are better only up to a point where the velocity is still significantly higher than zero to actually act as a gene drive.

Our results also point to an important role for dispersal in mediating outcomes. In our simulations (and those of others; Champer, Kim, et al., 2021), higher dispersal rates led to lower failure probability, and this occurred well below dispersal rates that would be consistent with panmixis. Closer examination revealed that this result is driven by a different scaling between dispersal rate and the widths of WT and drive waves. Drive wave width appears to scale super‐linearly with dispersal, whereas WT waves are linear or sub‐linear. Thus, for a given increase in dispersal, drive waves get wider disproportionately faster than WT waves (i.e. the wave gets wider faster, not the velocity). The precise reason for this remains unknown, but likely rests with the advection and asymmetric gene flow that emerges along the trailing edge of the drive wave. In the meantime, it seems that many of the spatial causes of drive failure (Figure 1) become negligible for species in which dispersal distances are very large relative to the scale of local dynamics.

Given both the promise and risk of suppression gene drives, their dynamics are worthy of careful examination. Since most populations exhibit spatial structure, finite population sizes and bounded ranges, these considerations are important in the overall design of a drive system. Our results very clearly demonstrate that a system optimized assuming an aspatial scenario is quite likely a long way from optimal in a spatially explicit scenario. In parallel with solving the molecular genetic challenges in developing suppression gene drive constructs in model and non‐model species, we also need to study novel gene drive systems under stochastic and spatially explicit scenarios. Doing so can very quickly make a promising candidate (e.g. X‐shredder) appear far from promising. Ongoing work in this space will clearly benefit from close collaboration between geneticists, evolutionary biologists and ecologists.

## AUTHOR CONTRIBUTIONS

BP conceived the study and wrote the initial framework of the simulations. JP developed and finalized the simulation framework. JP and BP performed the analyses, drafted, edited, and finalized the manuscript.

## CONFLICT OF INTEREST

The authors declare no conflict of interest.

## Supporting information


Appendix S1

## Data Availability

The code and the simulation output (.Rds R data format) are available in a public GitHub repository: https://github.com/jeffersonfparil/drivechasingecol.
