# Supplementary material for: Slow and steady wins the race: Spatial and stochastic processes and the failure of suppression gene drives
Source: Mol Ecol. 2022 Jul 22;31(17):4451–64. doi: 10.1111/mec.16598 (PMC9541681; doi:10.1111/mec.16598)
Supplement: Supplementary file 1 — Appendix S1 [file MEC-31-4451-s001.pdf]

9 Supplementary Information

9.1 Derivation of W-shredder equations

In ZW/ZZ sex determination systems, *e.g.*, birds, reptiles, and lepidopteran pests, where ZW are females and ZZ are males.

$$Z^d W \xrightarrow{\text{c}} Z^d \tag{19}$$

Let:

- $P(\text{shredded W} \mid \text{drive}) = c$, i.e., the probability of shredding the W chromosome given drive be c;
- $P(\text{intact W} \mid \text{drive}) = 1 - c$, i.e., the probability of intact W chromosome given drive be $1 - c$;
- $P(\text{shredded Z} \mid \text{drive}) = 0$, i.e., the Z chromosome be unaffected by the W-shredding;
- $P(\text{intact Z} \mid \text{drive}) = 1$, i.e., the Z chromosome be intact;
- f be female;
- m be male;
- $P(d) = P(d \mid m) = P(d \mid f) = q$, i.e., the probability of the drive allele in the Z chromosome be q;

- $P(D) = P(D \mid m) = P(D \mid f) = 1 - q$, i.e., the probability of the wild-type or non-drive allele in the Z chromosome be $1 - q$.

Probability of Z and W chromosomes per genotype and per sex

Female sex chromosomes:

$$\begin{aligned} P(W \mid Z^d W \mid f) &= \frac{P(\text{intact W} \mid \text{drive})}{P(\text{intact W} \mid \text{drive}) + P(\text{intact Z} \mid \text{drive})} \\ &= \frac{1-c}{2-c} \end{aligned} \tag{20}$$

$$\begin{aligned} P(Z \mid Z^d W \mid f) &= \frac{P(\text{intact Z} \mid \text{drive})}{P(\text{intact W} \mid \text{drive}) + P(\text{intact Z} \mid \text{drive})} \\ &= \frac{1}{2-c} \end{aligned} \tag{21}$$

$$P(W \mid ZW \mid f) = 1/2 \tag{22}$$

$$P(Z \mid ZW \mid f) = 1/2 \tag{23}$$

$$\begin{aligned} P(W \mid f) &= P(d \mid f)\ P(W \mid Z^d W \mid f) + P(D \mid f)\ P(W \mid ZW \mid f) \\ &= q\frac{1-c}{2-c} + (1-q)\frac{1}{2} = \frac{2-c-cq}{4-2c} \end{aligned} \tag{24}$$

$$\begin{aligned} P(Z \mid f) &= P(d \mid f)\ P(Z \mid Z^d W \mid f) + P(D \mid f)\ P(Z \mid ZW \mid f) \\ &= q\frac{1}{2-c} + (1-q)\frac{1}{2} = \frac{2-c+cq}{4-2c} \end{aligned} \tag{25}$$

Male sex chromosomes:

$$
\begin{aligned}
P(\text{W chromosome} \mid ZZ \mid m) &= P(\text{W chromosome} \mid ZZ^d \mid m) \\
&= P(\text{W chromosome} \mid Z^dZ \mid m) \\
&= P(\text{W chromosome} \mid Z^dZ^d \mid m) \\
&= 0
\end{aligned} \tag{26}
$$

$$
\begin{aligned}
P(\text{Z chromosome} \mid ZZ \mid m) &= P(\text{Z chromosome} \mid ZZ^d \mid m) \\
&= P(\text{Z chromosome} \mid Z^dZ \mid m) \\
&= 1
\end{aligned} \tag{27}
$$

$$
P(\text{W chromosome} \mid m) = 0 \tag{28}
$$

$$
P(\text{Z chromosome} \mid m) = 1 \tag{29}
$$

898 **Probability of offspring genotypes at the drive locus in the Z chro-**
899 **mosome**

900 Female genotypes:

$$
\begin{aligned}
P(D0) = P(Z^DW) &= P(D \cap Z \mid m) \times P(\text{W chromosome} \mid f) \\
&= P(D \mid m) \times P(\text{Z chromosome} \mid m) \times P(\text{W chromosome} \mid f) \\
&= (1-q) \times 1 \times \frac{2-c-cq}{4-2c} = \frac{2-c-2q+cq^2}{4-2c}
\end{aligned} \tag{30}
$$

$$
\begin{aligned}
P(d0) = P(Z^d W) &= P(d \cap Z \mid m) \times P(W \mid f) \\
&= P(d \mid m) \times P(Z \mid m) \times P(\text{W chromosome} \mid f) \\
&= q \times 1 \times \frac{2-c-cq}{4-2c} \\
&= \frac{2q-cq-cq^2}{4-2c}
\end{aligned}
\tag{31}
$$

901 Male genotypes:

$$
\begin{aligned}
P(DD) = P(Z^D Z^D) &= P(D \cap Z \mid m) \times P(D \cap Z \mid f) \\
&= P(D \mid m) \times P(Z \mid m) \times P(D \mid f) \times P(Z \mid ZW \mid f) \\
&= (1-q) \times 1 \times (1-q) \times 1/2 \\
&= \frac{1-2q+q^2}{2}
\end{aligned}
\tag{32}
$$

$$
\begin{aligned}
P(Dd) = P(Z^D Z^d) &= P(D \cap Z \mid m) \times P(d \cap Z \mid f) \\
&= P(D \mid m) \times P(Z \mid m) \times P(d \mid f) \times P(Z \mid Z^d W \mid f) \\
&= (1-q) \times 1 \times q \times \frac{1}{2-c} \\
&= \frac{q-q^2}{2-c}
\end{aligned}
\tag{33}
$$

$$
\begin{aligned}
P(dD) = P(Z^d Z^D) &= P(d \cap Z \mid m) \times P(D \cap Z \mid f) \\
&= P(d \mid m) \times P(Z \mid m) \times P(D \mid f) \times P(Z \mid ZW \mid f) \\
&= q \times 1 \times (1-q) \times 1/2 \\
&= \frac{q-q^2}{2}
\end{aligned}
\tag{34}
$$

$$
\begin{aligned}
P(dd) &= P(Z^d Z^d) = P(d \cap Z \mid m) \times P(d \cap Z \mid f) \\
&= P(d \mid m) \times P(Z \mid m) \times P(d \mid f) \times P(Z \mid Z^d W \mid f) \\
&= q \times 1 \times q \times \frac{1}{2-c} \\
&= \frac{q^2}{2-c}
\end{aligned} \tag{35}
$$

Frequency of the drive allele in the Z chromosome of the offspring

$$
\begin{aligned}
P(d \mid offspring) &= \frac{P(Dd)/2 + P(dD)/2 + P(dd) + P(d0)}{P(DD) + P(Dd) + P(dD) + P(dd) + P(D0) + P(d0)} \\
&= \frac{\frac{q-q^2}{2(2-c)} + \frac{q-q^2}{2\times 2} + \frac{q^2}{2} + \frac{2q-cq-cq^2}{4-2c}}{\frac{1-2q+q^2}{2} + \frac{q-q^2}{2-c} + \frac{q-q^2}{2} + \frac{q^2}{2} + \frac{2-c-2q+cq^2}{4-2c} + \frac{2q-cq-cq^2}{4-2c}} \\
&= \frac{\frac{8q-3cq-cq^2}{4(2-c)}}{1} \\
&= \frac{8q-3cq-cq^2}{8-4c}
\end{aligned} \tag{36}
$$

In other words:

$$
q_{t+1} = \frac{8q_t - 3cq_t - cq_t^2}{8-4c} \tag{37}
$$

Derivative of drive allele frequency with respect to time

If we were to assume overlapping generations (continuous time), then the derivative with respect to time can be expressed as:

$$
\begin{aligned}
\frac{dq}{dt} &= \frac{q_{t+1} - q_t}{t + 1 - t} \\
&= \frac{8q_t - 3cq_t - cq_t^2}{8 - 4c} - q_t \\
&= \frac{cq(1-q)}{8 - 4c},
\end{aligned} \tag{38}
$$

and the solution can be expressed as:

$$
q(t) = \frac{q_0 e^{ct/(8-4c)}}{1 + q_0(e^{ct/(8-4c)} - 1)} \tag{39}
$$

This can be thought of as an approximation recursive equation q_{t+1} above.

Population suppression

Population suppression is proportional to the frequency of females. The frequency of females in the next generation is equivalent to the frequency of the W chromosome:

$$
\begin{aligned}
P(f)_{t+1} &= P(\text{W chromosome} \mid f) \\
&= \frac{2 - c - cq}{4 - 2c}
\end{aligned} \tag{40}
$$

The population size in the next generation, N_{t+1} can be computed as the frequency of the females, $P(f)$ multiplied by the population size in the current generation, N_t and the fecundity of each female which we define

as the Beverton-Holt growth rate, $r_t = R_0/(1 + \alpha n_t)$, where $n_t = \frac{N_t}{\text{area}}$ is the population density, $\alpha = (R_{\max} - 2)/2N^*$ where $R_{\max}$ is the maximum fecundity and N^* is the carrying capacity given $R_{\max}$:

$$\begin{aligned} N_{t+1} &= N_t \times P(f) \times r_t \\ &= N_t \left(\frac{2 - c - cq_{t-1}}{4 - 2c} \right) \left(\frac{R_{\max}}{1 + n_t \frac{R_{\max}-2}{2N^*}} \right). \end{aligned} \tag{41}$$

9.2 Derivation of X-shredder equations

Re-derivation or a verbose derivation with inputs from Deredec et al. 2008:

In XX/XY sex determination systems, *e.g.*, mammals, where XX are females and XY are males.

$$XY^d \xrightarrow{\text{c}} 0Y^d \tag{42}$$

Let:

- $P(\text{shredded X} \mid \text{drive}) = c$, i.e., the probability of shredding the X chromosome given drive be c;

- $P(\text{intact X} \mid \text{drive}) = 1 - c$, i.e., the probability of intact X chromosome given drive be $1 - c$;

- $P(\text{shredded Y} \mid \text{drive}) = 0$, i.e., the Y chromosome be unaffected by the X-shredding;

- $P(\text{intact Y} \mid \text{drive}) = 1$, i.e., the Y chromosome be intact;
- $P(d) = P(d \mid m) = q$, i.e., the probability of the drive allele in the Y chromosome (in males) be q; and
- $P(D) = P(D \mid m) = 1 - q$, i.e., the probability of the wild-type or non-drive allele in the Y chromosome (in males) be $1 - q$.

Probability of X and Y chromosomes per genotype and per sex

Female sex chromosomes:

$$\begin{aligned} P(X \mid f) &= P(X \mid XX) \\ &= 1 \end{aligned} \tag{43}$$

$$\begin{aligned} P(Y \mid f) &= P(Y \mid XX) \\ &= 0 \end{aligned} \tag{44}$$

Male sex chromosomes:

$$\begin{aligned} P(X \mid XY^d \mid m) &= \frac{P(\text{intact X} \mid \text{drive})}{P(\text{intact X} \mid \text{ X shredding}) + P(\text{intact Y} \mid \text{drive})} \\ &= \frac{1-c}{2-c} \end{aligned} \tag{45}$$

$$P(Y \mid XY^d \mid m) = \frac{P(\text{intact Y} \mid \text{X shredding})}{P(\text{intact X} \mid \text{X shredding}) + P(\text{intact Y} \mid \text{drive})} = \frac{1}{2-c} \tag{46}$$

$$P(X \mid XY^D \mid m) = 1/2 \; P(Y \mid XY^D \mid m) = 1/2 \tag{47}$$

$$\begin{aligned} P(X \mid m) &= P(D \mid m) \times P(X \mid XY^D \mid m) + P(d \mid m) \times P(X \mid XY^d \mid m) \\ &= q\frac{1-c}{2-c} + (1-q)\frac{1}{2} \\ &= \frac{2-c-cq}{4-2c}. \end{aligned} \tag{48}$$

$$\begin{aligned} P(Y \mid m) &= P(D \mid m) \times P(Y \mid XY^D \mid m) + P(d \mid m) \times P(Y \mid XY^d \mid m) \\ &= P(Y^D) + P(Y^d) \\ &= (1-q)\;\frac{1}{2} + q\;\frac{1}{2-c} \\ &= \frac{2-c+cq}{4-2c}. \end{aligned} \tag{49}$$

938 **Probability of offspring genotypes in the drive allele locus (i.e., in**
939 **males only)**

$$\begin{aligned} P(XY^D) &= P(X \mid f) \times P(Y^D \mid m) \\ &= P(X \mid f) \times P(D \mid m) P(\text{Y chromosome} \mid XY^D \mid m) \\ &= (1) \times (1-q)\frac{1}{2} \\ &= \frac{1-q}{2}. \end{aligned} \tag{50}$$

$$
\begin{aligned}
P(XY^d) &= P(X \mid f) \times P(Y^d \mid m) \\
&= P(X \mid f) \times P(d \mid m) P(\text{Y chromosome} \mid XY^d \mid m) \\
&= (1) \times q \left(\frac{1}{2-c} \right) \\
&= \frac{q}{2-c}.
\end{aligned} \tag{51}
$$

940 **Frequency of the drive allele in the Y chromosome of the offspring**

$$
\begin{aligned}
P(d \mid \text{Y chromosome})_{t+1} &= \frac{P(XY^d)}{P(XY^D) + P(XY^d)} \\
&= \left(\frac{q}{2-c} \right) \left(\frac{1-q}{2} + \frac{q}{2-c} \right)^{-1} \\
&= \frac{2q}{2-c+cq}.
\end{aligned} \tag{52}
$$

941 In other words,

$$
q_{t+1} = \frac{2q}{2-c+cq}. \tag{53}
$$

942 If we were to assume overlapping generations (continuous time), then its
943 derivative with respect to time can be expressed as:

$$
\begin{aligned}
\frac{dq}{dt} &= \frac{q_{t+1} - q_t}{t+1-t} \\
&= \frac{2q_t}{2-c+cq_t} - q_t \\
&= \frac{cq(1-q)}{2-c+cq}.
\end{aligned} \tag{54}
$$

Population suppression

Population suppression is proportional to the frequency of females. The frequency of males in the next generation is equivalent to the frequency of the Y chromosome:

$$\begin{aligned} P(males)_{t+1} &= P(\text{Y chromosome} \mid m) \\ &= \frac{2 - c + cq}{4 - 2c}, \end{aligned} \tag{55}$$

then the frequency of females in the next generation is:

$$\begin{aligned} P(females)_{t+1} &= 1 - P(males) \\ &= 1 - \frac{2 - c + cq}{4 - 2c} \\ &= \frac{2 - c - cq}{4 - 2c}. \end{aligned} \tag{56}$$

This is exactly the same as in W-shredder. Hence, the population size in the next generation can be expressed as:

$$\begin{aligned} N_{t+1} &= N_t \times P(female) \times r_t \\ &= N_t \left(\frac{2 - c - cq_{t-1}}{4 - 2c} \right) \left(\frac{R_{\max}}{1 + n_t \frac{R_{\max} - 2}{2N^*}} \right). \end{aligned} \tag{57}$$

.

9.3 Supplementary Figures

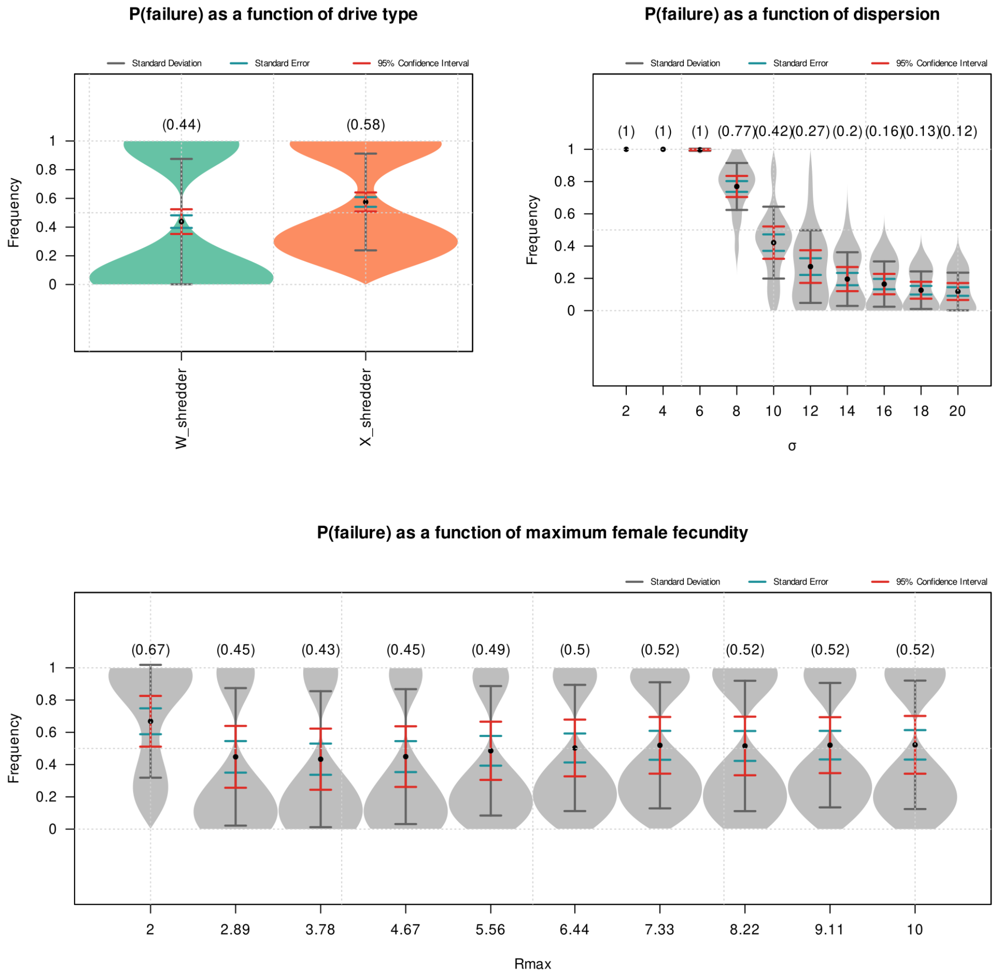

Supplementary Figure 1: Violin plots of the frequency of suppression gene drive failure as a function of drive type, dispersion parameter (Sigma), and maximum female fecundity (Rmax).

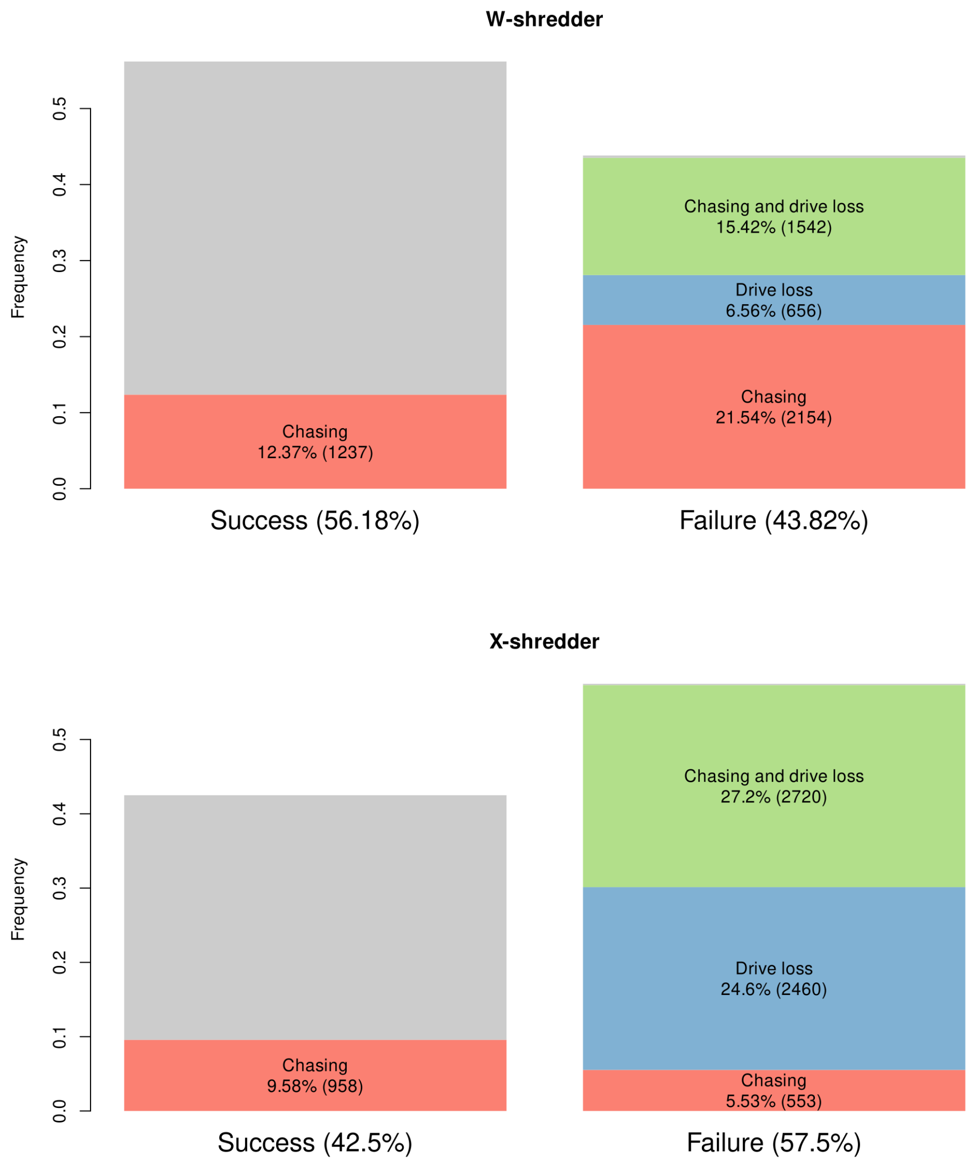

Supplementary Figure 2: Frequencies of the simulation outcomes (suppression gene drive success and failure), and their intersections with the events that can cause failure:loss of suppression gene drive allele and chasing in W-shredder and X-shredder drive systems.

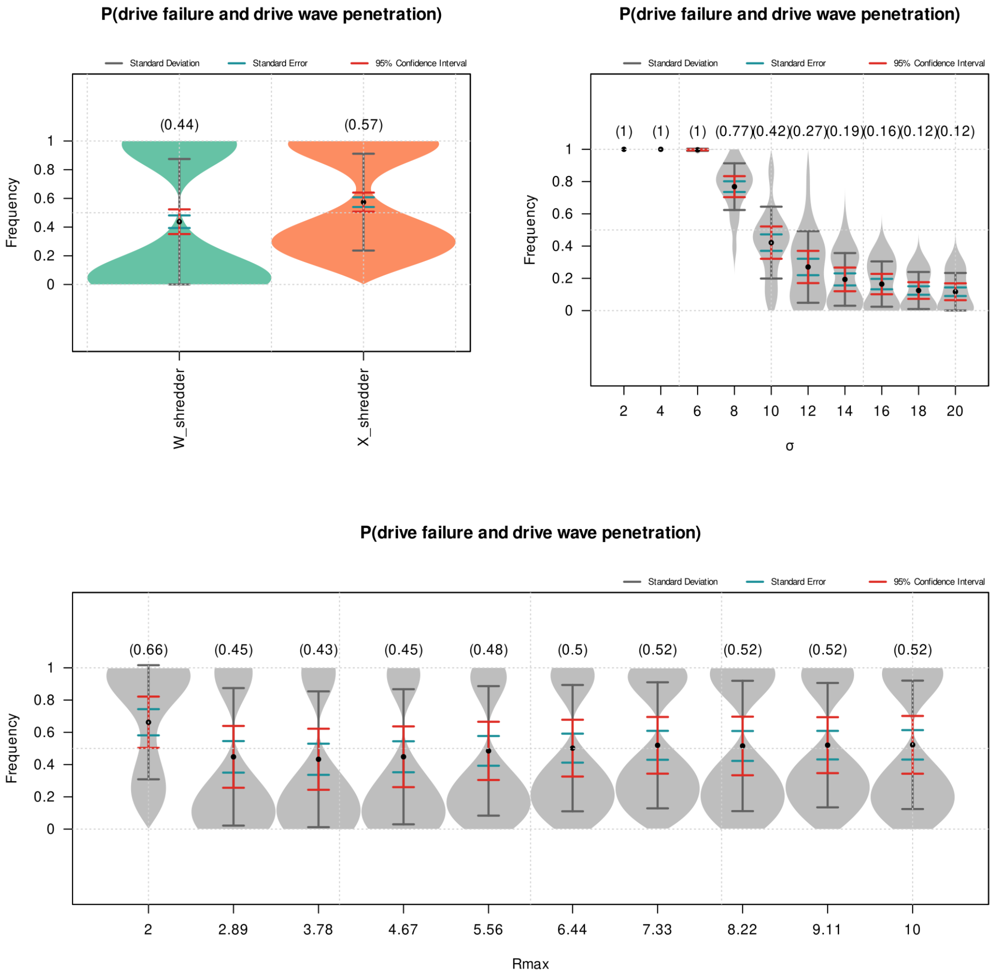

Supplementary Figure 3: Violin plots of the frequency of suppression gene drive failure due to drive wave penetration as a function of drive type, dispersion parameter (Sigma), and maximum female fecundity (Rmax).

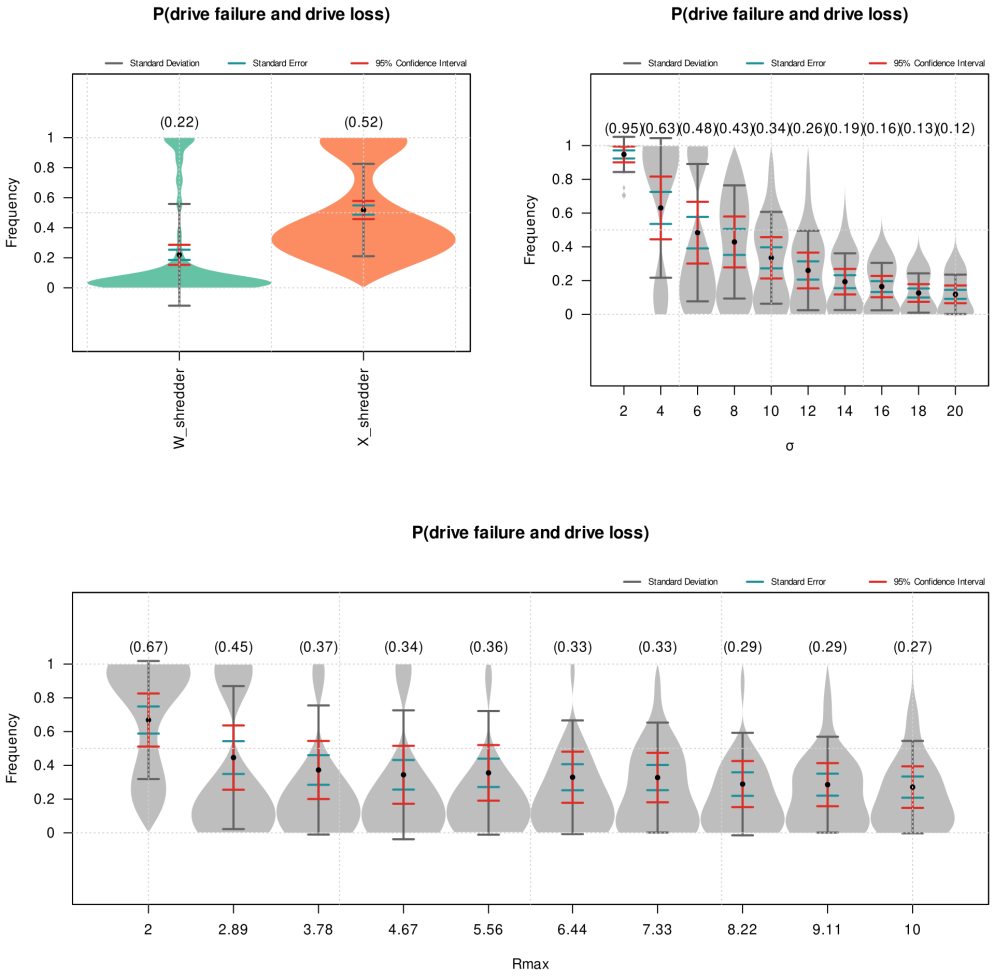

Supplementary Figure 4: Violin plots of the frequency of suppression gene drive failure due to the loss of the drive allele as a function of drive type, dispersion parameter (Sigma), and maximum female fecundity (Rmax).

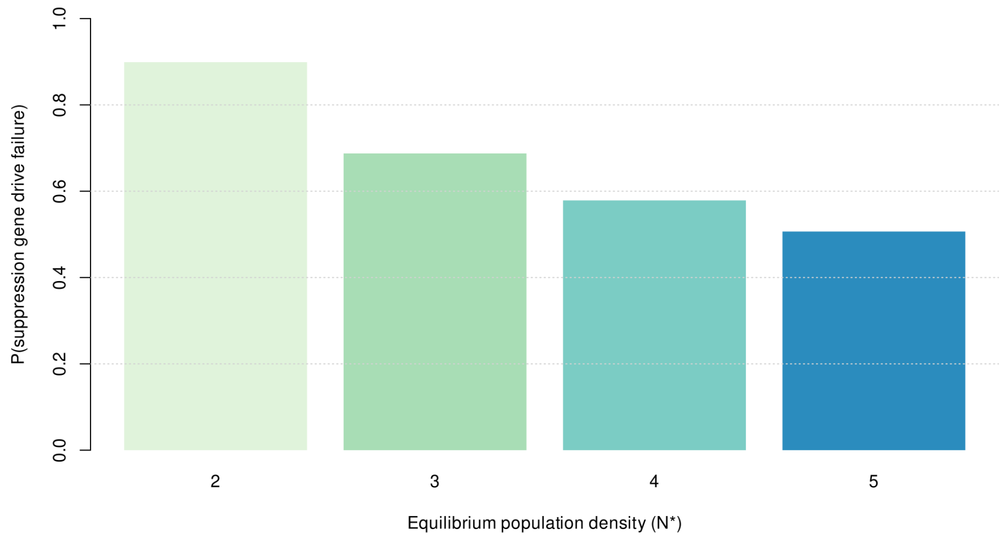

Supplementary Figure 5: Barplot of the frequency of suppression gene drive failure as a function of the equilibrium population density (N^*).
